# Oncogenic and Receptor-Mediated Wnt Signaling Influence the Sensitivity of Colonic Cells to Butyrate

**DOI:** 10.7150/jca.82393

**Published:** 2023-02-05

**Authors:** Michael Bordonaro

**Affiliations:** Department of Medical Education, Geisinger Commonwealth School of Medicine, 525 Pine Street, Scranton, PA 18509, USA

**Keywords:** Wnt signaling. Butyrate, oncogenic, receptor-mediated, APC

## Abstract

Deregulated Wnt signaling is responsible for most cases of colorectal cancer (CRC). Dietary fiber is protective against CRC and this activity is likely mediated by butyrate, a breakdown product of dietary fiber that hyperactivates Wnt signaling, repressing CRC proliferation and inducing apoptosis. Receptor-mediated Wnt signaling and oncogenic Wnt signaling, which is typically initiated by mutation in more downstream elements of the pathway, activate non-overlapping patterns of gene expression. Receptor-mediated signaling is associated with a poor prognosis for CRC while oncogenic signaling is associated with a relatively good prognosis. We have compared the expression of genes differentially expressed in receptor-mediated vs. oncogenic Wnt signaling to microarray data generated in our laboratory. Most importantly we evaluated these gene expression patterns comparing the early stage colon microadenoma line LT97 with the metastatic CRC cell line SW620. LT97 cells exhibit a gene expression pattern more strongly associated with that observed with oncogenic Wnt signaling, while SW620 cells exhibit a gene expression pattern moderately associated with that observed with receptor-mediated Wnt signaling. Given that SW620 cells are more advanced and malignant compared to LT97 cells, these findings are generally consistent with the better prognosis observed with tumors exhibiting a more oncogenic Wnt gene expression pattern. Importantly, LT97 cells are more sensitive to the effects of butyrate on proliferation and apoptosis that are CRC cells. We further examine these gene expression patterns in butyrate-resistant vs. butyrate-sensitive CRC cells. Based upon all of these observations, we hypothesize that colonic neoplastic cells exhibiting a more oncogenic as compared to receptor-mediated Wnt signaling gene expression pattern would be more sensitive to the effects of butyrate, and, hence, fiber, than are those cells exhibiting a more receptor-mediated Wnt signaling pattern of expression. Diet-derived butyrate may affect the differential patient outcomes resulting from the two types of Wnt signaling. We further posit that development of butyrate resistance and concomitant changes in Wnt signaling patterns, including associations with CBP and p300, disrupts the association between the two major types of Wnt signaling (receptor-mediated and oncogenic) and neoplastic progression/prognosis. Ideas about testing the hypothesis and therapeutic implications are briefly considered.

## Introduction

Deregulated Wnt signaling is responsible for most cases of colorectal cancer (CRC) 1-3. Dietary fiber is protective against CRC and this activity is likely mediated by butyrate, a breakdown product of dietary fiber that hyperactivates Wnt signaling, repressing CRC proliferation and inducing apoptosis 4-6. However, individuals may still develop CRC despite a high fiber diet; thus, resistance to the anti-CRC action of butyrate likely contributes to colonic tumorigenesis. To evaluate butyrate resistance we have developed a butyrate-resistant CRC cell line (HCT-R) 6 and have analyzed gene expression in this line compared to butyrate-sensitive parental HCT-116 CRC cells 7.

The histone acetylases CREB Binding Protein (CBP) and p300 activate different sets of Wnt target genes and therefore have different phenotypic consequences for cells 8. Thus, Wnt activity mediated by CBP is typically associated with cancer cell proliferation; in contrast Wnt signaling influenced by p300 has been associated with differentiation 8. HCT-R cells exhibit downregulation of p300 expression 9,10, and we have shown that p300 knockout HCT-116 cells are partially butyrate-resistant and that restoration of p300 expression 11 in such cells can resensitize them to the effects of butyrate 12. CBP and p300 compete for binding to the Wnt factor beta-catenin and hence increased CBP-Wnt activity comes at the expense of p300-Wnt activity, and *vice versa* 8. Thus, it is likely that CRC cells deficient in p300 have increased CBP-Wnt activity and, hence, more proliferation and viability in the presence of butyrate (butyrate-resistance) 12.

Using a human colon organoid approach, it was shown that the gene expression profile resulting from oncogenic (*APC* KO) Wnt signaling is markedly different from that induced by receptor-mediated signaling 13. The oncogenic Wnt signaling is associated with better patient prognosis and a consensus molecular subtype 2 (CMS2) tumor, even though CRCs remain “addicted” to Wnt activity 13. It is uncertain why oncogenic Wnt activity is associated with better prognosis; one possibility is that this activity is associated with early adenomas and more advanced tumors downregulate Wnt signaling, but on the other hand, CRCs can remain “Wnt addicted” and in some cases Wnt signaling is associated with invasiveness 13. Perhaps, as the authors suggest, it is the specific downstream responses to the signaling, rather the level of the signaling itself, that is more important. Thus, the responsiveness of different Wnt target genes to Wnt signaling may change over the lifetime of a neoplasm, modified by environmental context. On the other hand, receptor-mediated Wnt signaling, associated with CMS4 tumors, correlates to a worse prognosis 13.

Genes modulated for the receptor-mediated signaling pathway include *ADAMTS14, ASIC1, SLC2A3,* and* SMOC1*; genes modulated for the oncogenic pathway include *ATOH8, BMX, CKB, FCGBP, ID3, MTMR11, RA12,* and* VAV3* 13. It is useful to examine the expression of the abovementioned sets of genes in microarray data generated in our laboratory 7,14,15, in order to better understand the association between receptor vs. oncogenic Wnt signaling and colonic neoplasia, which can inform understanding and hypothesis about how this association may be affected by dietary fiber/butyrate.

*SLCA3* and *SMOC1* are among the genes whose expression is upregulated by butyrate in a Wnt-signaling dependent manner in HCT-116 cells, while the upregulation of expression of *ATOH8* by butyrate is partially dependent upon Wnt activity 14. In those cases, the Wnt activity is oncogenic, but due to *beta-catenin*, rather than *APC,* mutation. And in all cases, the array studies performed using a physiologically relevant concentration (5 mM) of butyrate.

**Fig 1:**
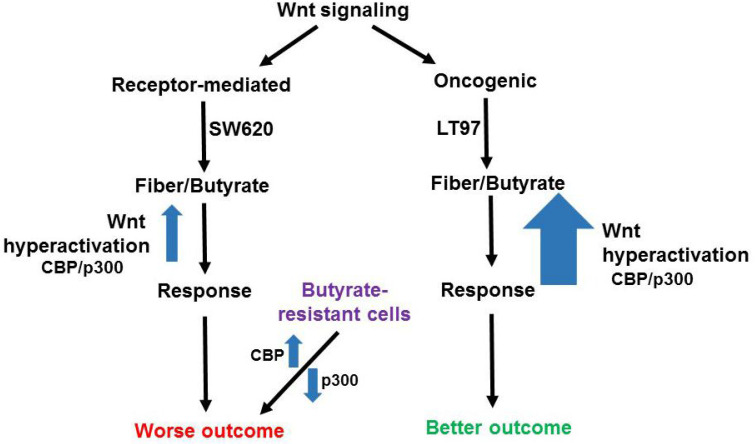
** Potential effects of butyrate on oncogenic and receptor-mediated gene expression profiles in neoplastic colonic cells**. Canonical Wnt signaling can be broadly divided between receptor-mediated Wnt signaling (left) and oncogenic Wnt signaling (right), activated by mutations in APC or beta-catenin. The former signaling is represented by a gene expression pattern more similar to that of the metastatic SW620 CRC cell line and results in a worse outcome for CRC patients, while the latter type of signaling is similar to that of LT97 adenoma cells and results in a better outcome in CRC. LT97 cells are more sensitive to the effects of butyrate, derived from dietary fiber, than are CRC cells. Thus, we posit that oncogenic Wnt signaling results, possibly mediated by CBP/p300, can be hyperactivated to a greater degree than receptor-mediated signaling, leading to a cell response (less proliferation and more apoptosis) associated with better outcomes. While the better outcomes for patients may be independent of Wnt modulation by butyrate, we note that physiological levels of butyrate in the colonic lumen reach and surpass that which can result in the aforementioned responses in cell culture. Therefore, it is possible that diet-derived butyrate affects the outcomes for patients with tumors with different Wnt signaling gene expression patterns. Butyrate-resistant cells have disrupted CBP-Wnt and p300-Wnt signaling and this results in worse outcomes independent of possessing a typical receptor-mediated Wnt signaling gene expression pattern. In this case, the loss of p300, and the consequent butyrate-resistance, obviates the requirement for other elements of a receptor-mediated molecular signature.

Most important is the comparison of gene expression between SW620 metastatic CRC cells and the early-stage microadenoma line LT97 16. Consistent with the idea that a receptor-mediated gene expression profile correlates to a worse prognosis, both *ADAMTS14* and *SLC2A3* are more highly expressed in SW620 cells than in LT97 cells (+/- butyrate); however, *SMOC1* unexpectedly exhibits the opposite pattern 15. The situation for the oncogenic Wnt signaling pattern, which is correlated to a better prognosis, is more clear-cut. Thus, *CKB, FCGBP, ID3, MTMR11*, and *VAV3* are all more highly expressed in early-stage LT97 adenoma cells (+/- butyrate) compared to metastatic SW620 cells, while *ATOH8* is more highly expressed in LT97 cells but only in the absence of butyrate 15. Therefore, this is a clear pattern of the better-prognosis oncogenic Wnt gene expression pattern being associated with benign adenoma cells as compared to metastatic carcinoma cells, and this association holds in the presence of butyrate with the exception of *ATOH8*. It should be noted that both SW620 and LT97 cells exhibit oncogenic Wnt signaling due to *APC* mutation, similar to the colon organoids of the Michels et al. study 13, and it is important to note that LT97 cells are particularly sensitive to the effects of butyrate on cell proliferation and apoptosis compared to CRC cells such as SW620 cells 15,17 (Fig. 1).

HCT-R cells are a butyrate-resistant form of HCT-116 CRC cells, and comparing HCT-R to HCT-116 gene expression 7 yields a mixed gene expression pattern with respect to receptor-mediated vs oncogenic Wnt signaling molecular profiles. *ADAMTS14* is downregulated in HCT-R cells compared to HCT-116 cells, while *SLC2A3* shows the opposite pattern (both +/- butyrate). *ATOH8* is upregulated and *FCGBP* is downregulated in HCT-R cells compared to HCT-116 cells (both +/- butyrate), while *CKB* is downregulated in HCT-R cells in the presence of butyrate, compared to HCT-116 cells. HCT-R cells exhibit downregulates expression of p300, which can be a cofactor in Wnt signaling, and we have shown that this downregulation is associated with butyrate resistance 12,15. HCT-116 cells engineered to be p300 knockout (KO) are partially butyrate-resistant, and the gene expression patterns of such cells have been compared to the parental HCT-116 line 12. Expression of *ADAMTS14* is downregulated in the KO cells, as is expression of *ASIC1*, while *MTMR11* is modestly upregulated (not statistically significant). One reason why the data for the butyrate resistant cell line vs. HCT-116 comparisons are not as clear-cut as that of SW620 vs. LT97 is that while SW620 cells are clearly more advanced along the neoplastic spectrum than are LT97, the situation for the butyrate-resistant lines are not as clear. On the one hand, loss of p300 can be associated with worse prognosis [11, 12 and refs. therein]; p300 KO lines show increased migration in culture 11, and resistance to butyrate is also reasonably seen as leading to worse outcomes. On the other hand, HCT-R cells tend to proliferate less rapidly than parental HCT-116 cells, and the *in vivo* tumorigenic potential of HCT-R vs. HCT-116 cells has not yet been evaluated. It is possible that butyrate-resistant cells, which have disrupted response of Wnt signaling to butyrate, with decreased p300 responses, exhibit a disrupted association between the two major types of Wnt signaling (receptor-mediated and oncogenic) and neoplastic progression/prognosis (Fig. 1). This deficiency in p300 would tip the balance in favor of more CBP-Wnt activity, more proliferation, and a worse prognosis, even though these butyrate-resistant cells would not mimic the same receptor-mediated Wnt gene expression profile as, e.g., SW620 cells (Fig. 1). We also note that *ADAMTS14* and *ASIC1* are downregulated in p300 KO cells 12, demonstrating that p300 deletion can induce a butyrate-resistant phenotype even without some of the gene expression profiles associated with receptor-mediated Wnt signaling.

Increased expression of *ADAMTS14*, which codes for a metalloproteinase that can affect the extracellular matrix, and of *SLC2A3,* a glucose transporter, could conceivably promote tumorigenesis through effects on the tumor microenvironment and on metabolism, consistent with the association of receptor-mediated Wnt gene expression with a poorer prognosis. Interestingly, expression of the transcription factor *ATOH8* has been associated with poor survival in CRC and knockdown of the gene inhibits cancer cell proliferation 18, which is not consistent with the oncogenic pathway being related to improved outcomes, although the effects of the expression of specific genes may be dependent on context, such as the stage of neoplasia. Similarly *CKB* is associated with cancer progression, yet is part of the oncogenic Wnt pathway. This counter-intuitive pattern continues with *FCGBP*, associated with metastasis and poor outcomes in CRC 19, and *VAV3*, the expression of which correlates to poor outcomes in CRC and can promote cancer cell growth 20. *ID3* is associated with higher prostate cancer grade.

The researchers 13 also found that the *APC* KO organoid cells exhibit limited changes in gene expression when exposed to the extrinsic stimulus of receptor-mediated signaling, suggesting that in this system, canonical Wnt signaling is relatively saturated by the *APC* KO status. It is unknown how the organoid *APC* KO system would react to stimulation by HDACis such as butyrate. While upregulation of outside-in receptor-mediated signaling, which is still active in CRC cells with Wnt-activating mutations, is one mechanism whereby HDACis hyperactive Wnt signaling 6, other mechanisms, including enhanced binding of Tcf-beta-catenin complexes to target DNA, also contribute to the effect 21. Furthermore, butyrate modulates Wnt signaling through effects of the histone acetylases CBP and p300 9,10. Therefore, even if increased receptor-mediated signaling could not contribute to Wnt hyperactivation in the *APC* KO organoid cell model, these other mechanisms may be able to affect Wnt signaling levels. Evaluating response of the organoid models to butyrate (and other HDACis) would constitute part of the approach to test the hypothesis (below).

Detailed clinical, cellular, biochemical, and molecular comparisons between oncogenic and receptor-mediated Wnt signaling, with respect to the issues covered in this manuscript, are summarized in Table 1.

## Hypothesis

We hypothesize that colonic neoplastic cells exhibiting a more oncogenic as compared to receptor-mediated Wnt signaling gene expression pattern would be more sensitive to the effects of butyrate, and, hence, fiber, than are those cells exhibiting a more receptor-mediated Wnt signaling pattern of expression. This main hypothesis allows us to derive further, consequent hypotheses. Diet-derived butyrate may affect the differential patient outcomes resulting from the two types of Wnt signaling. Thus, levels of butyrate in the colonic lumen would hyperactivate Wnt signaling and induce apoptosis to a greater degree in patients with tumors exhibiting more of an oncogenic Wnt signaling gene expression pattern than those with tumors of the receptor-mediated type. These differences could decrease tumor load, and affect tumor grade, probability of metastasis, as well as response to treatment. It is possible that differences in the types of tumors (e.g., CMS2 vs. CMS4) that are found in patients are affected by dietary history. Thus, a high fiber diet, with higher levels of colonic butyrate, may select against the development of CMS2 tumors (oncogenic signaling that is more sensitive to butyrate) as opposed to CMS4 tumors (more resistant receptor-mediated signaling). Thus, development of CRC in the context of a high fiber diet may not only be due to butyrate resistance *per se*, but also to a particular molecular signature that is naturally less sensitive to the effects of butyrate. Given that CMS4 tumors tend to have a worse prognosis, a high fiber diet would have the advantage of reducing overall risk of CRC, but when CRC does develop, it may have a worse prognosis. We further posit that development of butyrate resistance and concomitant changes in Wnt signaling patterns, including associations with CBP and p300, disrupts the association between the two major types of Wnt signaling (receptor-mediated and oncogenic) and neoplastic progression/prognosis.

## Testing the hypothesis

**Fig 2:**
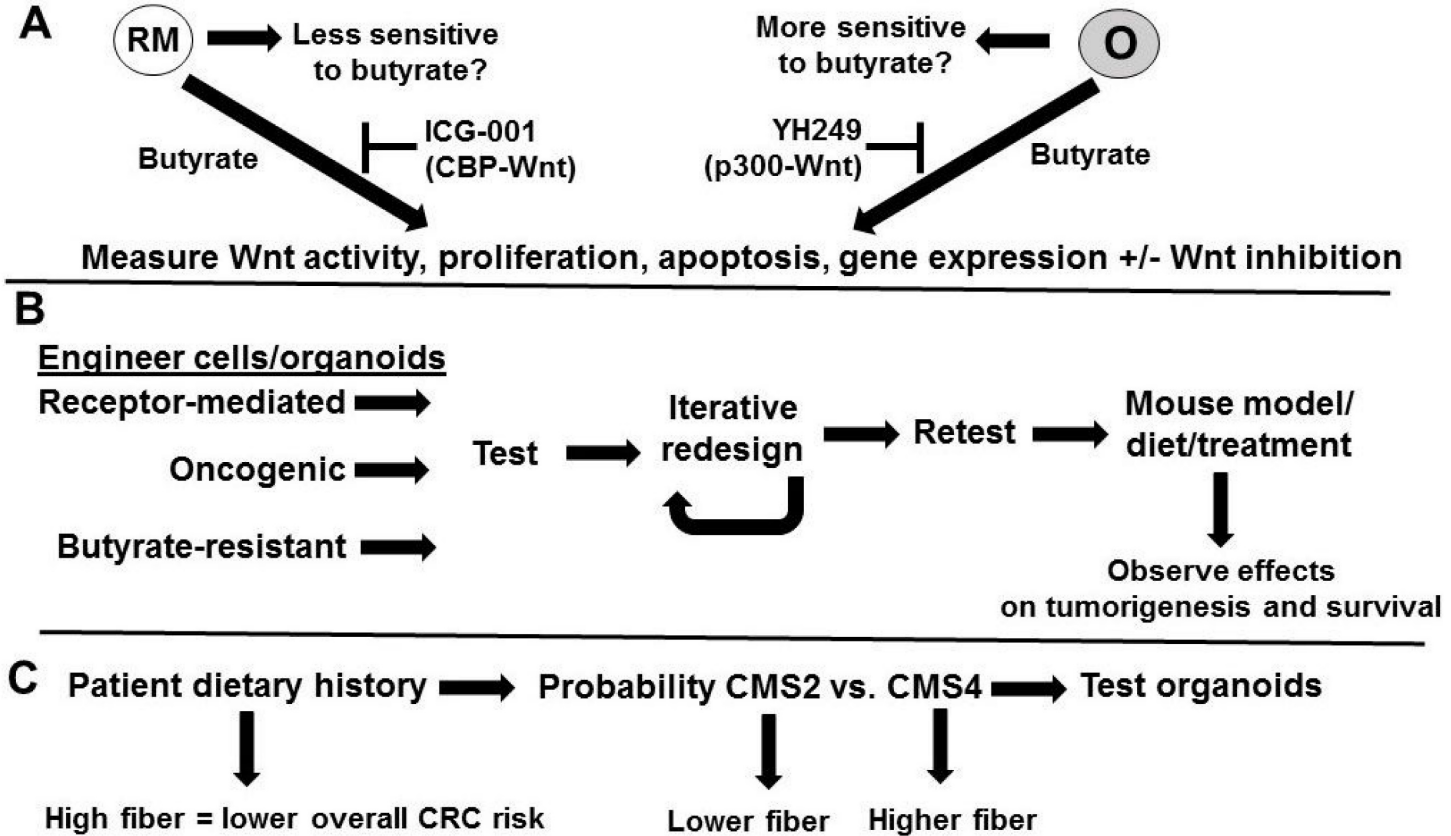
** Testing the hypothesis.** (A) Testing the hypothesis that organoids with oncogenic (0) Wnt signaling will be more sensitive to butyrate than those with receptor-mediated (RM) signaling and that this will be at least in part mediated by CBP/p300. The two types of organoids 13 will be treated with butyrate and metrics such as Wnt signaling, proliferation, apoptosis, and the expression of genes diagnostic of the two molecular signatures 13 will be determined. Dependence of Wnt activity for observed effects will be determined through the use of a Wnt inhibitor. Repressing CBP-Wnt activity with ICG-001 may enhance the ability of butyrate to induce apoptosis in RM cells, while repressing p300-Wnt signaling with YH249 may induce butyrate resistance in O cells, although potentiation of butyrate effects are theoretically possible through p300-independent mechanisms 23. (B) Engineering cells to mimic molecular signatures and consequent phenotypes and further evaluation *in vivo*. Cells will be modified to reflect gene expression patterns similar to that found in receptor-mediated Wnt signaling organoids, oncogenic Wnt signaling organoids, or in butyrate-resistant cells. Resulting cells will be tested as in (A) and an iterative approach will be used to redesign the cells to reflect the optimized phenotypes expected for each of these cell types. These cells could then be implanted in nude mouse models, and tumorigenesis and survival assayed under different dietary conditions or with treatment with butyrate. (C) Relationship between patient dietary history and tumor type. We would expect high fiber diets to be associated with overall lower CRC risk, but for those patients with CRC, high fiber diets may select for CMS4 tumors with receptor-mediated Wnt signaling molecular signatures and a poor prognosis. Organoids can be prepared from patient tumor samples and evaluated as in ref. 13 and in (A)

The hypothesis can be tested by three approaches (Fig. 2). First, the hypothesis that organoids with oncogenic Wnt signaling will be more sensitive to butyrate than those with receptor-mediated signaling can be evaluated (Fig. 2A) by treating the two types of organoids 13 with butyrate, and metrics such as Wnt signaling, proliferation, apoptosis, and the expression of genes diagnostic of the two molecular signatures can be determined. Dependence of Wnt activity for the observed effects can be determined through the use of a Wnt inhibitor, such as iCRT3. The hypothesis that differences in CBP-Wnt vs. p300-Wnt signaling may in part mediate the relative efficacy of butyrate in the two types of cells can be tested utilizing specific inhibitors of those signaling pathways. Thus, repressing CBP-Wnt activity with ICG-001 8 may enhance the ability of butyrate to induce apoptosis in cells that favor receptor-mediated Wnt signaling, while repressing p300-Wnt signaling with YH249 22 may induce butyrate resistance in cells that favor oncogenic Wnt signaling, although data from our laboratory suggests that, in some cases, YH249 can potentiate the effects of butyrate on colonic cells 23. However, these latter effects are most likely independent of p300, Wnt activity, and p300-Wnt activity 23, and distinguishing between Wnt-dependent and Wnt-independent effects of YH249 can be determined through the use of the more general Wnt inhibitor iCRT3.

CBP or p300 knockout (e.g., CRISPR) can also be utilized, although one would expect a greater number of (possibly off-target) effects by a general knockout of these factors, as opposed to a more targeted pathway inhibition with the pharmacological agents. However, use of Wnt inhibitors can be used to better define the Wnt signaling-dependent components of the effects of CBP or p300 knockout, with ICG-001 more consistently mimicking CBP ablation 8 than YH249 does for p300 23.

Next, the hypothesis can be evaluated *in vivo*; however, first genetic engineering of CRC cells should be performed to ascertain what components of the receptor-mediated vs. oncogenic Wnt signaling molecular signatures are required for effects of butyrate treatment and to optimize response or resistance to the effects of butyrate treatment. Thus, CRC cells can be modified to reflect gene expression patterns similar to that found in receptor-mediated Wnt signaling organoids, oncogenic Wnt signaling organoids, or in butyrate-resistant cells. Cells can be stably transfected with expression vectors for the relevant genes, and CRISPR can be used to knockout other relevant genes, depending on whether increased or decreased expression is desired. The gene expression patterns used to model these engineered cells would be derived from ref. 13, as well as from microarray data from our laboratory 7,14,15 as well as that of other researchers.

The resulting cells could then be tested according to the same metrics outlined above and an iterative approach used to redesign the cells to reflect the optimized phenotypes expected for each cell type. This approach could be used to evaluate the extent that CBP-Wnt vs. p300-Wnt signaling is involved in the differential outcomes of receptor-mediated Wnt signaling vs. oncogenic Wnt signaling, particularly in conjunction with butyrate treatment. CBP-Wnt signaling would be expected to be relatively more important in neoplasia that is derived more from receptor-mediated Wnt signaling, while the oncogenic Wnt signaling profile would be expected to exhibit a relatively greater reliance on p300 and p300-Wnt activity. Knockout and/or overexpression of these two factors may be an important component of the iterative process of engineering cells to express the optimized molecular signatures and cell phenotypes characteristic of the organoid types studied in ref. 13.

The optimized cells could then be implanted in nude mouse models, and tumorigenesis and survival assayed under different dietary conditions or with treatment with butyrate. One would expect that cells with an “extreme” receptor-mediated Wnt molecular signature would be butyrate resistant, similar to cells designed to be butyrate-resistant (e.g., with a molecular signature similar to the HCT-R line), and would be more aggressively tumorigenic in animal models. Conversely, cells with optimized oncogenic Wnt molecular profiles would be more sensitive to butyrate, and less tumorigenic in mouse models. Dietary interventions leading to increased levels of colonic butyrate would be more effective in orthotopic nude mouse models in which oncogenic Wnt signaling-optimized tumors are implanted into the colon, as opposed to receptor mediated Wnt signaling-optimized tumors.

Finally, the relationship between patient dietary history and tumor type can be ascertained (Fig. 2C). According to our hypothesis, we would expect high fiber diets to be associated with overall lower CRC risk, but for those patients with CRC, high fiber diets may select for CMS4 tumors with receptor-mediated Wnt signaling molecular signatures and poor prognosis. Organoids can be prepared from patient tumor samples and tested as in ref. 13 and in Fig. 2A. We would expect that these organoids would recapitulate the previously observed molecular signatures and physiological characteristic observed in the original study 13 and in the experiments proposed above. Further, it may be possible that the CMS4 tumors selected for in high fiber diet contexts (assuming that such selection occurs) would exhibit more extreme molecular signatures and phenotypes, compared to that generally observed in patients not stratified by diet 13. One may expect that CMS4 tumors from patients with very high fiber diet histories to possess characteristics that to some extent mimic those exhibited by the cells intentionally engineered to exhibit an extreme receptor-mediated Wnt signaling molecular signature and phenotype, suggestive of selective pressure on neoplastic cells to evade the effects of high concentrations of butyrate in the colonic lumen.

## Conclusion

While dietary fiber is protective against CRC, likely in part due to the activity of butyrate, resistance to butyrate would decrease the preventive efficacy of a high fiber diet 2,3,6. The differential patterns of gene expression resulting from receptor-mediated and oncogenic Wnt signaling, associated with worse and better CRC prognosis respectively, may exhibit variable sensitivity to butyrate. We hypothesize that oncogenic Wnt signaling exhibits a greater sensitivity to, and hyperactivation by, butyrate than does receptor-mediated signaling, resulting in a cell response leading to a better prognosis (e.g., less proliferation and more apoptosis. These differences are represented by cell culture models; hence, the adenoma cell line LT97 exhibits a greater degree of oncogenic Wnt expression than do metastatic SW620 CRC cells and the LT97 cells are, not surprisingly, more sensitive to butyrate than are CRC cells [15,17).

The degree of Wnt hyperactivation exhibited by the different Wnt signaling pathways may be mediated by effects of CBP and p300 on Wnt activity and consequent gene expression. Therefore, we expect that different classes of CRC, representing greater or lesser degrees of receptor-mediated vs. oncogenic Wnt signaling will exhibit differential sensitivity to butyrate and to therapeutic HDACis, with clinical significance. This may be amenable to modulation by specific inhibitors of CBP-Wnt vs. p300-Wnt activity 8,22. Butyrate-resistant cells are deficient in p300 expression 10,12 and this may disrupt the association between outcome and type (receptor-mediated vs. oncogenic) Wnt signaling. Hence, butyrate-resistant cells would represent tumors with poor outcomes despite having gene expression profiles that overlap that of SW620-type and LT97-type cell lines (Fig. 1). It is possible that diet-derived butyrate, mediated through effects on Wnt signaling and cell physiology, is in part responsible for the differential effects on outcome/patient prognosis for the type types of Wnt signaling. These hypotheses can be tested (Fig. 2) and have possible clinical implications for CRC prevention and therapy. This testing is of particular value given findings of the importance of various signaling pathways in CRC 24-30, including those that influence Wnt signaling 25,26, 28-30. These other signaling pathways can be included in the categories of (a) receptor-mediated Wnt signaling and/or (b) those related to butyrate resistance 27, and the non-Wnt signaling pathways may exhibit cross-talk with Wnt activity, particularly Wnt activity that is mediated by CBP and p300.

Finally, we note the relevance and novel aspects of this work compared to the general field of butyrate studies on CRC, as well as our own work in this field. While there has been much focus on the role of butyrate in mediating the preventive effects of fiber against CRC, our work on this topic has been unique in relating these effects of butyrate to the fundamentally important Wnt signaling pathway. The first observation that the Wnt signaling pathway can be pharmacologically modulated, particularly by histone deacetylase inhibitors (including butyrate), and the physiological implications of this for CRC prevention, was from the work of the present author, in the Augenlicht laboratory 4. Subsequently, details of the molecular mechanisms of this modulation, including gene expression profiles and the role of CBP/p300, were outlined by our group 2,3,5-7, 9-10,12. The novel aspects of the current manuscript compared to our previous work is the finding that cellular, biochemical, and molecular aspects of butyrate's effects on different types of colonic cells correlate to oncogenic vs. receptor-mediated Wnt signaling, which has clinical relevance given the differences in prognosis of CRCs that emphasize one form of that signaling over the other. Further, we have extended this clinical relevance by determining that early adenoma cells combine aspects of oncogenic Wnt signaling with a greater response to butyrate, while metastatic SW620 CRC cells exhibit more characteristics of receptor-mediated Wnt signaling and less responsiveness to butyrate, possibly correlated to a poorer prognosis. Finally, we present a novel and testable hypothesis, based upon the relationship between the development of butyrate resistance and all of the aforementioned novel aspects of this manuscript. This hypothesis may assist in understanding how those CRCs that develop despite a high fiber diet may be selected to be more butyrate resistant and may possess certain characteristics (e.g., a CMS4 sub-type phenotype) leading to a worse clinical prognosis. These findings and the hypothesis may lead to fruitful future work that can target prevention of CRCs with worse clinical outcomes.

## Figures and Tables

**Table 1:** ** Summary of clinical, cellular, biochemical, and molecular characteristics of oncogenic and receptor-mediated Wnt signaling**. The table emphasizes the comparison between LT97 and SW620 cells, as well as the relevance to fiber/butyrate.

Type of Wnt Signaling	Oncogenic	Receptor-Mediated
**Clinical characteristics**	Better clinical outcomes, but possibly can have increased invasiveness as well; CMS2 tumor subtype	Worse clinical outcomes; CMS4 tumor subtype
**Cellular characteristics**	LT97-like (early adenoma); *APC* KO organoids; more butyrate-sensitive but can become butyrate-resistant (HCT-R cells are partially in this category); selected against by a high fiber diet	SW620-like (more advanced, including metastatic); in general, more butyrate-resistant resistant (HCT-R cells are partially in this category); is more likely if CRC develops despite a high fiber diet
**Biochemical**	Less likely to exhibit receptor-mediated Wnt signaling; more dependent on oncogenic mutations (“Wnt addicted”); fewer receptor-ligand interactions driving Wnt activity-mediated cell growth; more responsive to butyrate with respect to Wnt signaling activation	Typically more strongly dependent on receptor-mediated Wnt signaling in addition to oncogenic mutations; receptor-ligand interactions (particularly those feeding into Wnt signaling) important; less Wnt signaling activation upon exposure to butyrate
**Molecular**	Typically, expression of *ATOH8, BMX, CKB, FCGBP, ID3, MTMR11, RA12,* and* VAV3*; specifically, *CKB, FCGBP, ID3, MTMR11*, and *VAV3* are more highly expressed (+/- butyrate) in LT97 adenoma cells, and *ATOH8* is more highly expressed in LT97 cells (absence of butyrate only)	Typically, expression of *ADAMTS14, ASIC1, SLC2A3,* and* SMOC1*; specifically, *ADAMTS14* and *SLC2A3* are more highly expressed (+/- butyrate) in SW620 cells; cell phenotype possibly mimicked by p300 deletion (albeit without same gene expression pattern)

## Abbreviations

APC: Adenomatous polyposis coli
KO: knockout
HDACi: histone deacetylase inhibitor
CBP: CREB Binding Protein
